# Inhibition of Voltage-Gated Na^+^ Currents Exerted by KB-R7943 (2-[2-[4-(4-nitrobenzyloxy)phenyl]ethyl]isothiourea), an Inhibitor of Na^+^-Ca^2+^ Exchanging Process

**DOI:** 10.3390/ijms24021805

**Published:** 2023-01-16

**Authors:** Sheng-Nan Wu, Meng-Cheng Yu

**Affiliations:** 1 Department of Physiology, National Cheng Kung University Medical College, Tainan 70101, Taiwan; 2 Institute of Basic Medical Sciences, National Cheng Kung University Medical College, Tainan 70101, Taiwan; 3 Department of Post-Baccalaureate Medicine, National Sun Yat-sen University Medical College, Kaohsiung 80708, Taiwan

**Keywords:** KB-R7943, Na^+^-Ca^2+^ exchange, voltage-gated Na^+^ current, transient Na^+^ current, late Na^+^ current, window Na^+^ current, resurgent Na^+^ current, persistent Na^+^ current, current kinetics

## Abstract

KB-R7943, an isothiourea derivative, has been recognized as an inhibitor in the reverse mode of the Na^+^-Ca^2+^ exchanging process. This compound was demonstrated to prevent intracellular Na^+^-dependent Ca^2+^ uptake in intact cells; however, it is much less effective at preventing extracellular Na^+^-dependent Ca^2+^ efflux. Therefore, whether or how this compound may produce any perturbations on other types of ionic currents, particularly on voltage-gated Na^+^ current (*I*_Na_), needs to be further studied. In this study, the whole-cell current recordings demonstrated that upon abrupt depolarization in pituitary GH_3_ cells, the exposure to KB-R7943 concentration-dependently depressed the transient (*I*_Na(T)_) or late component (*I*_Na(L)_) of *I*_Na_ with an IC_50_ value of 11 or 0.9 μM, respectively. Likewise, the dissociation constant for the KB-R7943-mediated block of *I*_Na_ on the basis of a minimum reaction scheme was estimated to be 0.97 μM. The presence of benzamil or amiloride could suppress the *I*_Na(L)_ magnitude. The instantaneous window Na^+^ current (*I*_Na(W)_) activated by abrupt ascending ramp voltage (V_ramp_) was suppressed by adding KB-R7943; however, subsequent addition of deltamethrin or tefluthrin (Tef) effectively reversed KB-R7943-inhibted *I*_Na(W)_. With prolonged duration of depolarizing pulses, the *I*_Na(L)_ amplitude became exponentially decreased; moreover, KB-R7943 diminished *I*_Na(L)_ magnitude. The resurgent Na^+^ current (*I*_Na(R)_) evoked by a repolarizing V_ramp_ was also suppressed by adding this compound; moreover, subsequent addition of ranolazine or Tef further diminished or reversed, respectively, its reduction in *I*_Na(R)_ magnitude. The persistent Na^+^ current (*I*_Na(P)_) activated by sinusoidal voltage waveform became enhanced by Tef; however, subsequent application of KB-R7943 counteracted Tef-stimulated *I*_Na(P)_. The docking prediction reflected that there seem to be molecular interactions of this molecule with the hNa_V_1.2 or hNa_V_1.7 channels. Collectively, this study highlights evidence showing that KB-R7943 has the propensity to perturb the magnitude and gating kinetics of *I*_Na_ (e.g., *I*_Na(T)_, *I*_Na(L)_, *I*_Na(W)_, *I*_Na(R)_, and *I*_Na(P)_) and that the Na_V_ channels appear to be important targets for the in vivo actions of KB-R7943 or other relevant compounds.

## 1. Introduction

The Na^+^-Ca^2+^ (NCX) exchanger is recognized to be an important regulator of intracellular Ca^2+^ concentration that is expressed in cardiac sarcolemma but also in brain, skeletal muscle, and endocrine tissues [1,2,3,4,5,6]. KB-R7943 (2-[2-[4-(4-nitrobenzyloxy)phenyl]ethyl]isothiourea) is an isothiourea derivative which is thought to selectively inhibit the reverse mode of NCX isoform 1 (NCX1) with effective IC_50_ of 1.2–2.4 μM [7]. This compound was demonstrated to prevent intracellular Na^+^-dependent Ca^2+^ uptake in intact cells [7]; however, it is much less effective at preventing extracellular Na^+^-dependent Ca^2+^ efflux. Furthermore, in cultured hippocampal neurons, it has been reported to exhibit neuroprotection from glutamate-induced excitotoxicity by blocking NMDA receptor-mediated activity (IC_50_ = 13.4 μM) as well as by suppressing the activity of complex I in the mitochondrial respiratory chain (IC_50_ = 11.4 μM) [8]. KB-R7943 has also been shown to block transient receptor potential canonical channels, which are important modulators of Ca^2+^-dependent signal transduction [9]. One of the novel thiourea derivatives (i.e., 1-naphthalen-1-yl-3[5-(3-thioureido-phenoxy)-pentyl]-thiourea (compound #326)) has been previously demonstrated to modify different types of membrane ion channels [10]. Therefore, apart from its inhibition of the NCX exchanging process, the issue of how or whether KB-R7943 or other relevant compounds could exercise any modifications on plasmalemmal ionic currents has not yet been studied. The NCX activity could be indirectly altered by changes in the magnitude of voltage-gated Na^+^ current (*I*_Na_) [3,5,6]. Therefore, it is worthwhile to reappraise the ionic mechanism of KB-R7943 actions which may exist in different types of ionic currents, particularly at *I*_Na_, because significant modifications in magnitude and gating kinetics of this current have been recently investigated for their therapeutic or pharmacological effectiveness [11,12,13,14,15,16].

There are nine isoforms (i.e., Na_V_1.1-1.9 (or SCN1A-SCN5A and SCN8A-SCN11A)) of voltage-gated Na^+^ (Na_V_) channels which are distributed in mammalian excitable tissues that include the central or peripheral nervous system, and the neuroendocrine or endocrine system [17,18,19]. Upon being activated, the Na_V_ channel activity, which constitutes macroscopic *I*_Na_, is to briefly depolarize the membrane and to initiate or generate the upstroke of the action potential; consequently, changes in *I*_Na_ magnitude can control the firing amplitude, frequency, and patterns inherent in variable types of electrically excitable cells [18,19,20,21,22,23,24]. It also needs to be mentioned that several inhibitors of Na_V_ channels (e.g., ranolazine (Ran), sparsentan, mirogabalin, esaxerenone, and carbamazepine (CBZ)) have been recently demonstrated [25,26,27,28,29,30,31], while several activators of the channels (e.g., tefluthrin (Tef), deltamethrin (DLT), telmisartan, and apocynin) have been found to preferentially slow the inactivation rate as well as increase the late component of *I*_Na_ (*I*_Na(L)_) [22,32,33,34,35,36,37]. It is yet uncertain whether cell exposure to KB-R9743 or other relevant compounds (e.g., inhibitors of NCX exchanging process) can perturb the magnitude and gating kinetics of various types of *I*_Na_ (e.g., transient Na^+^ current (*I*_Na(T)_), late Na^+^ current (*I*_Na(L)_), window Na^+^ current (*I*_Na(W)_), resurgent Na^+^ current (*I*_Na(R)_), and persistent Na^+^ current (*I*_Na(P)_)).

Because of the initiatives described above, the hypothesis in this study is that KB-R7943 might cause direct perturbations on different types of transmembrane ionic currents, particularly on *I*_Na_, identified in pituitary GH_3_ cells. We also further evaluated the ionic mechanism of this compound through which it interacts with different types of *I*_Na_. Endocrine cells, including pituitary cells, have been previously demonstrated to express the presence of the NCX exchanging process [38,39,40,41,42,43]. Of importance, findings from these results provide evidence to show that the presence of KB-R7943 can directly cause a depressant action on *I*_Na_ in concentration-, time-, and state-dependent manners in these cells. The major depressant action of this compound on *I*_Na_ (e.g., *I*_Na(L)_, *I*_Na(W)_, *I*_Na(R)_, and *I*_Na(P_) demonstrated herein is thought to be direct and largely through its interaction with the open state (or conformation) of the Na_V_ channel.

## 2. Results

### 2.1. Suppressive Effect of KB-R7943 on Voltage-Gated Na^+^ Current (I_Na_) Identified from Pituitary GH_3_ Cells

**Figure 1:**
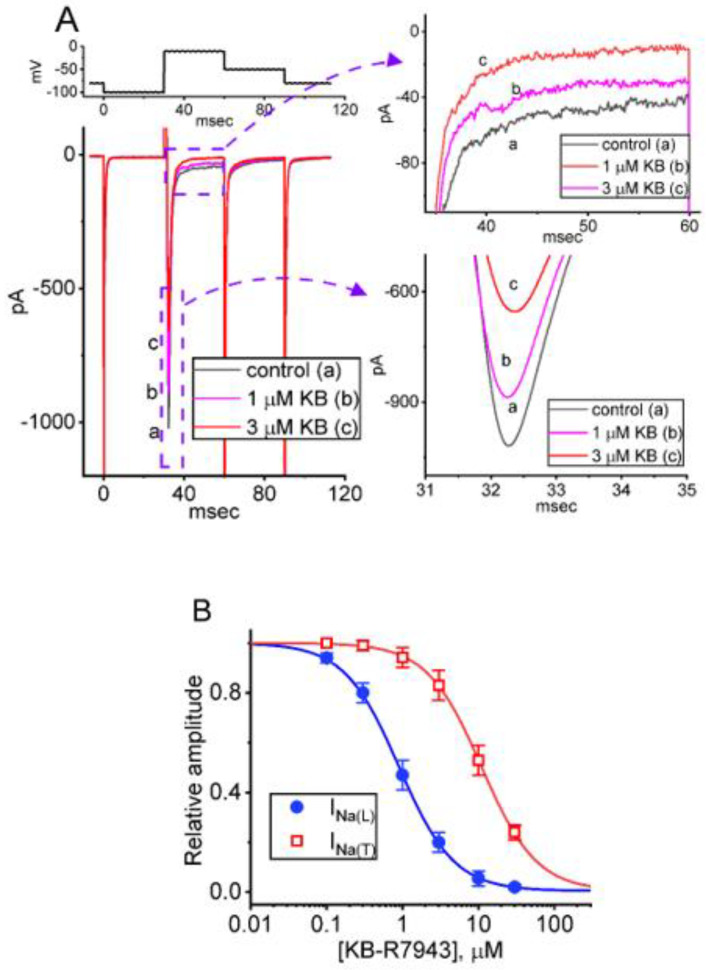
Inhibitory effect of KB−R7943 on voltage-gated Na^+^ current (*I*_Na_) identified from pituitary GH_3_ cells. The whole−cell current recordings were conducted in cells bathed in Ca^2+^−free Tyrode solution containing 10 mM tetraethylammonium chloride (TEA) and 0.5 mM CdCl_2_, and we filled up the measuring electrode with a solution containing Cs^+^. (**A**) Exemplar current traces obtained during control period (a, black color) and in the presence of 1 μM KB−R7943 (b, pink color, KB) or 3 μM KB−R7943 (c, red color, KB). The upper part shown the voltage clamp protocol given; graphs on the right side of (**A**) with dashed curve arrows show the expanded records from each purple broken box in the left side. (**B**) Concentration-response curve of KB−R7943−mediated inhibition of transient (peak) *I*_Na_ (*I*_Na(T)_) (open red squares) or late (sustained) *I*_Na_ (*I*_Na(L)_) (filled blue circles) observed in GH_3_ cells. The smooth blue or red line drawn represents the goodness-of-fit to the modified Hill equation, as elaborated in Section 4. The IC_50_ value for KB-R7943-induced inhibition of *I*_Na(T)_ or *I*_Na(L)_ seen in these cells was yielded to be 11 or 0.9 μM, respectively. Each point represents the mean ± SEM (*n* = 8−9).

In the initial stage of measurements, we explored if and how the presence of KB-R7943 could produce any perturbations on *I*_Na_ present in these cells. To prevent any contaminations by either the NCX exchanging process, voltage-gated Ca^2+^ currents, or Ca^2+^-induced inward currents [3,4,29,44,45], we placed cells in Ca^2+^-free Tyrode solution, and the measuring pipettes were filled with a solution enriched with Cs^+^. To evoke *I*_Na_, we voltage-clamped each tested cell at −80 mV. A hyperpolarizing pulse to −100 mV for a duration of 30 ms was then applied to precede the depolarizing command voltage from −100 to −10 mV, and such a depolarizing pulse was then given to activate *I*_Na_ (i.e., *I*_Na(T)_ and *I*_Na(L)_). Under these experimental conditions, we were able to detect the occurrence of a transient inward current (i.e., inward flux of cations) which displayed the rapidly activating and inactivating time course in the current (Figure 1A). Upon such a brief rectangular pulse, this type of transient inward current was sensitive to either suppression or stimulation by the presence of tetrodotoxin (TTX, 1 μM) or tefluthrin (Tef, 10 μM), respectively. However, neither cell exposure to nimodipine (1 μM) nor CdCl_2_ (0.5 mM) was able to alter the current magnitude. Therefore, it has been identified as a TTX-sensitive *I*_Na_ [22,28,34]. Furthermore, upon cell exposure to KB-R7943, the transient *I*_Na_ (*I*_Na(T)_) progressively became diminished in combination with the concurrent increase in inactivation rate of *I*_Na(T)_ (Figure 1A). For example, the application of KB-R7943 at a concentration of 1 or 3 μM KB-R7943 considerably decreased *I*_Na(T)_ amplitude to 887 ± 17 pA (*n* = 8, *p* < 0.05) or 693 ± 15 pA (*n* = 8, *p* < 0.05), respectively, from a control value of 983 ± 21 pA (*n* = 8). Concurrently, the time constant (τ_inact(S)_) in the slow component of *I*_Na(T)_ inactivation was accompanied by a significant reduction to 2.1 ± 0.3 ms (*n* = 8, *p* < 0.05) or 1.8 ± 0.2 ms (*n* = 8, *p* < 0.05), respectively, from a control value of 2.4 ± 0.3 ms (*n* = 8). However, no considerable changes in the time constant in the fast component of current inactivation were demonstrated with exposure to 1 or 3 μM KB-R7943. After KB-R7943 was removed, *I*_Na(T)_ amplitude was returned to 979 ± 21 pA (*n* = 8).

The sigmoidal relationship between the KB-R7943 concentration and the peak (*I*_Na(T)_) or late (*I*_Na(L)_) component of *I*_Na_ elicited by a short depolarization step from −100 to −10 mV was further constructed. As can be seen in Figure 1B, the cumulative application of KB-R7943 at the concentrations ranging between 0.1 and 30 μM resulted in a concentration-dependent reduction in the magnitude of both *I*_Na(T)_ and *I*_Na(L)_. According to a modified Hill equation described in Section 4, the IC_50_ value entailed for KB-R7943-induced suppression of *I*_Na(T)_ or *I*_Na(L)_ seen in GH_3_ cells was yielded to be 11 or 0.9 μM, respectively. Therefore, the results reflected that with exposure to this compound, the observed *I*_Na(L)_ magnitude was diminished to a greater extent than *I*_Na(T)_. Furthermore, in accordance with IC_50_ values, with the minimal reaction scheme stated in the Supplementary Information, the value of dissociation constant (*K*_D_) with the presence of KB-R7943 was calculated to be 0.97 μM, which was close to the IC_50_ for its inhibitory action on *I*_Na(L)_; however, the *K*_D_ value was lower than IC_50_ for its suppression on *I*_Na(T)_. The experimental observations allowed us to indicate that cell exposure to KB-R7943 concentration-dependently produced an inhibitory but differential effect on the magnitude of *I*_Na(T)_ and *I*_Na(L)_.

### 2.2. Comparison among Effects of Benzamil, Amiloride, Benzamil plus Tefluthrin (Tef), and Benzamil plus Deltamethrin (DLT) on I_Na(L)_ Amplitude Measured from GH_3_ Cells

**Figure 2:**
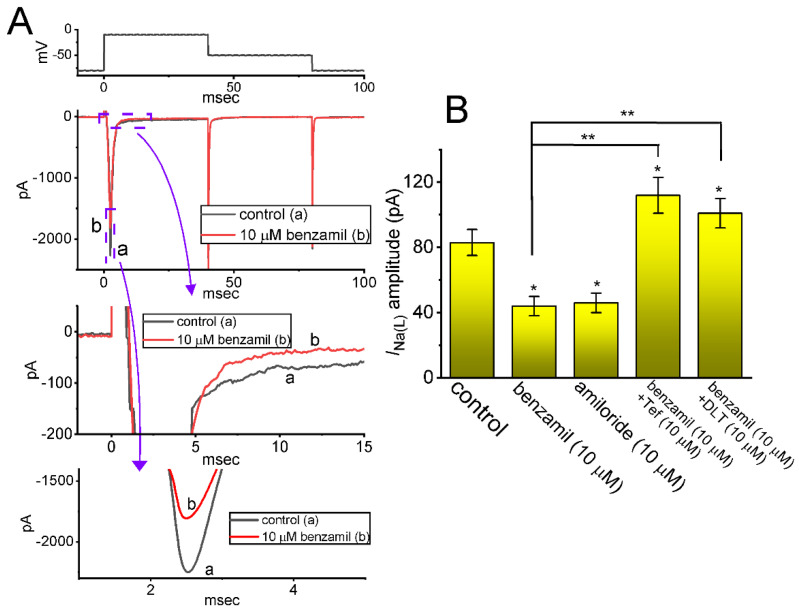
Effect of benzamil, amiloride, benzamil plus tefluthrin (Tef), and benzamil plus deltamethrin (DLT) on *I*_Na_ measured from GH_3_ cells. (**A**) Exemplar current traces elicited by rectangular depolarizing pulse from −80 to −10 mV for a duration of 40 ms. a: control (black color); b: in the presence of 10 μM benzamil (b, red color). The uppermost part is the voltage clamp protocol delivered, and the lower part of (**A**) shows the display of the expanded records in each purple dashed box. (**B**) Summary graph demonstrating effects of benzamil, amiloride, benzamil plus tefluthrin (Tef), and benzamil plus deltamethrin (DLT) on the amplitude of *I*_Na(L)_ (mean ± SEM; *n* = 7 for each yellow bar). The *I*_Na(L)_ amplitude was measured at the end of a short depolarizing pulse from −80 to −10 mV for a duration of 40 ms. * Significantly different from control (*p* < 0.05) and ** significantly different from benzamil (10 μM) alone group (*p* < 0.05).

We next explored if several other compounds (e.g., benzamil and amiloride) known to suppress the activity of NCX exchanging process could exert any modifications on the amplitude of *I*_Na_ in response to rapid membrane depolarization. Either benzamil or amiloride has been previously reported to suppress the activity of the NCX exchanging process, which is also present in pituitary cells [38,46]. Of note, as demonstrated in Figure 2, either further addition of benzamil (10 μM) or amiloride (10 μM) was able to diminish the amplitude of *I*_Na(T)_ or *I*_Na(L)_ in combination with a measurable raise in the inactivation time course (i.e., decrease in *I*_Na(T)_’s τ_inact(S)_). Moreover, with continued exposure to 10 μM benzamil, the subsequent addition of tefluthrin (Tef, 10 μM) or deltamethrin (DLT, 10 μM) was effective at reversing the benzamil-mediated decrease in *I*_Na(L)_ measured at the end-pulse of the short depolarizing step. Tef or DLT, which belongs to pyrethroid insecticides, has been reported earlier to be an activator of *I*_Na_ [22,29,32,34,36]. Therefore, cell exposure to benzamil or amiloride at a concentration of 10 μM can cause a reduction in *I*_Na(T)_ and *I*_Na(L)_ magnitude.

### 2.3. Inhibitory Effect of KB-R7943 on Average Steady-State Current Versus Voltage (I-V) Relationship of I_Na(T)_

**Figure 3:**
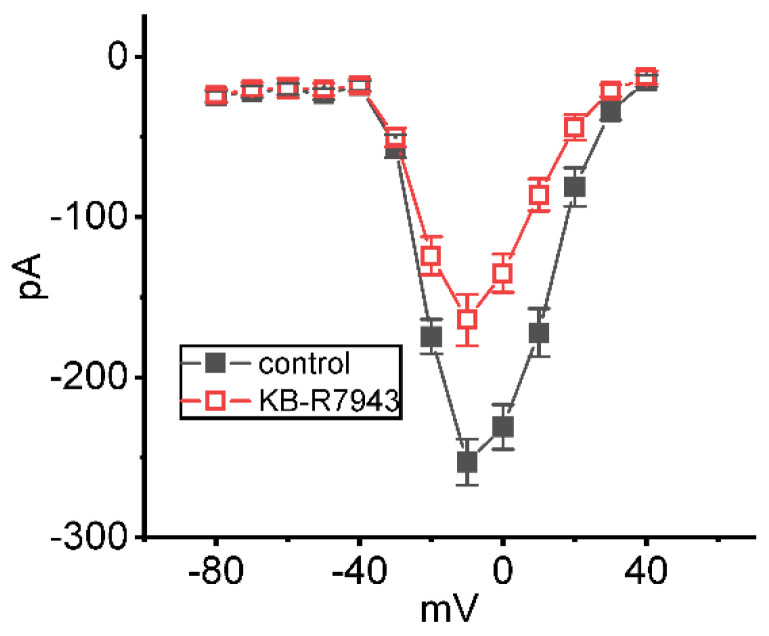
Effect of KB−R7943 on *I*_Na(T)_ evoked by different levels of depolarizing voltage commands measured from GH_3_ cells. Average current versus voltage (*I*−*V*) relationships of peak amplitude of *I*_Na(T)_ under control (filled black squares) and during the exposure to 3 μM KB-R7943 (open red squares). Each cell was depolarized from −80 mV to various potentials ranging from −80 to +40 mV in 10 mV increments for a duration of 30 ms. The *I*_Na(T)_ amplitude was measured at the beginning of each voltage pulse. Each point represents the mean ± SEM (*n* = 7).

In another separate set of measurements, we held the studied cells at −80 mV and then applied various levels of voltage pulses to them, from −90 to +40 mV in 10 mV increments for a duration of 30 ms. Under the experimental voltage protocols, a family of I_Na(T)_ could be robustly elicited and the currents were noticeably manifested by a rapid activating and inactivating property. Of note, one minute after cell exposure to 3 μM KB-R7943, the *I*_Na(T)_ magnitude became depressed, especially at the potentials ranging between −20 and +20 mV. Figure 3 depicts the *I-V* relationships (i.e., V-shaped configuration) of *I*_Na(T)_ measured at the beginning of each potential in the control period (i.e., absence of KB-R7943) and during exposure to 3 μM KB-R7943. The results hence showed that the overall quasi-steady-state *I-V* relationship of *I*_Na(T)_ remained unchanged with the presence of KB-R7943, despite its ability to decrease *I*_Na(T)_ amplitude.

### 2.4. Suppressive Effect of KB-R7943 on the Window Component of I_Na_ (I_Na(W)_) Measured from GH_3_ Cells

**Figure 4:**
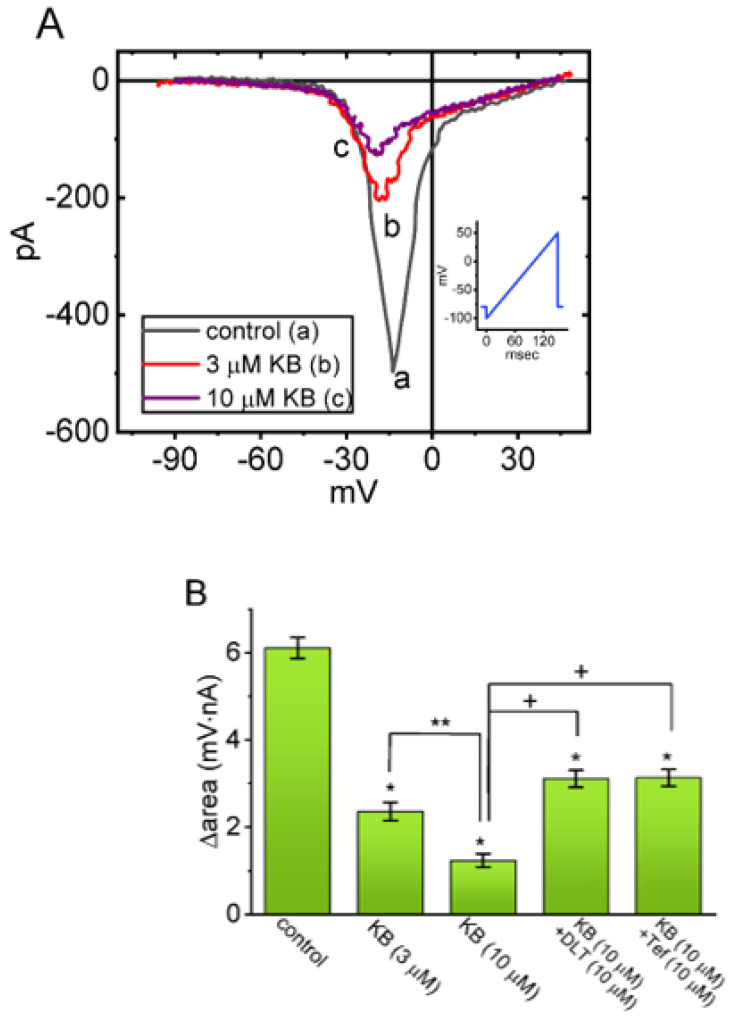
Inhibitory effect of KB-R7943 on nonlinear window *I*_Na_ (*I*_Na(W)_) activated by abrupt ascend−ng ramp voltage (V_ramp_) in GH_3_ cells. This set of whole−cell current recordings was undertaken with the tested cell voltage-clamped at −80 mV, and we then applied V_ramp_ from −100 to +50 mV for a duration of 150 ms on the cell. (**A**) Exemplar current traces were acquired during the control period (a, black color) and in the presence of 3 μM KB-R7943 (b, red color, KB) or 10 μM KB−R7943 (c, purple color, KB). The voltage clamp protocol applied is shown in the inset, whereas a downward deflection is indicated as the appearance of transient inward current (i.e., instantaneous *I*_Na(W)_) in response to short ascending V_ramp_. (**B**) Summary bar graph showing the effect of KB−R7943 (KB, 3 or 10 μM), KB-R7943 plus deltamethrin (DLT), and KB−R7943 plus tefluthrin (Tef) on the ∆area of *I*_Na(W)_ (mean ± SEM; *n* = 8 for each green bar). The ∆area of *I*_Na(W)_ (i.e., the relationship of membrane voltage versus current amplitude) was calculated at the area encircled under the voltages ranging between −90 and +40 mV during the short upsloping V_ramp_. * Significantly different from control (*p* < 0.05), ** significantly different from KB-R7943 (3 μM) alone group (*p* < 0.05), and ^+^ significantly different from KB−R7943 (10 μM) alone group (*p* < 0.05).

The presence of instantaneous *I*_Na(W)_ over the short period of time activated in response to the upsloping (or ascending) ramp voltage (V_ramp_) has been demonstrated earlier in varying excitable cells [28,29,47,48,49,50]. We thus proceeded to examine if the KB-R7943 presence could modify the magnitude of non-linear I_Na(W)_ evoked by abrupt ascending V_ramp_. To perform this separate set of measurements, we held the tested cell at −80 mV, and an ascending V_ramp_ from −100 to +50 mV for a duration of 150 ms (i.e., with a ramp speed of 1 mV/ms) was then imposed to activate instantaneous *I*_Na(W)_. Within one minute of exposing GH_3_ cells to KB-R7943 (3 or 10 μM), the strength (i.e., ∆area) of *I*_Na(W)_ induced by the 150 ms upsloping V_ramp_ was profoundly diminished (Figure 4A,B). For example, treating cells with 10 μM resulted in a striking reduction in *I*_Na(W)_’s ∆area from 6.11 ± 0.24 to 1.24 ± 0.15 mV·nA (*n* = 8, *p* < 0.05). Moreover, still with continued presence of 10 μM KB-R7943, the subsequent application of either Tef (10 μM) or DLT (10 μM) measurably attenuated the KB-R7943-mediated reduction in ∆area, as demonstrated by a marked elevation of ∆area value to 3.13 ± 0.19 mV·nA (*n* = 8, *p* < 0.05) or 3.11 ± 0.19 mV·nA (*n* = 8, *p* < 0.05), respectively. It is therefore clear from electrical recordings that the *I*_Na(W)_’s strength activated by the ascending V_ramp_ can be subject to being inhibited by the presence of KB-R7943.

### 2.5. KB-R7943-Mediated Slowing in Recovery from I_Na(L)_ either during Prolonged Duration of Depolarizing Pulse or by the Envelope-of-Tail Test

**Figure 5:**
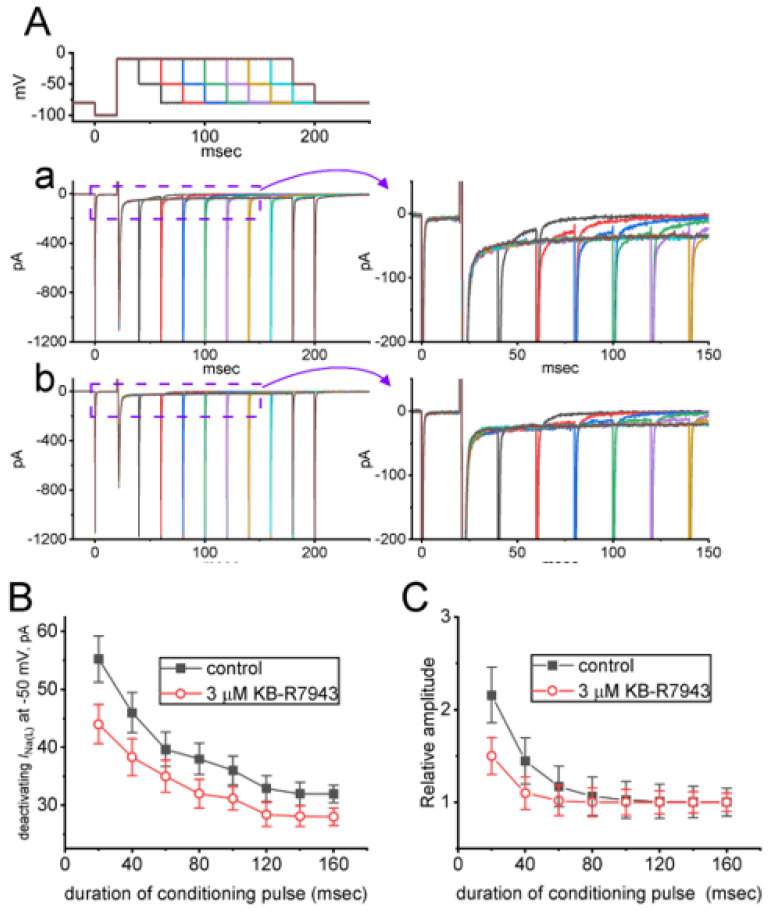
Effect of KB−R7943 on both recovery of *I*_Na(L)_ (**A**) and *I*_Na(L)_ magnitude evoked by the envelope−of−tail test (**B**) seen in GH_3_ cells. (**A**) Exemplar current traces taken during the control period (a, absence of KB-R7943) and with presence of 3 μM KB−R7943 (b). Different colors represent the specific current trace evoked by the voltage pulse with increasing duration at the level of −10 mV. The panels shown on the right side denote the expanded records from purple dashed boxes with solid curve arrows on the left side for better illustrations. (**B**) Relationship of the duration of depolarizing pulse versus the *I*_Na(L)_ amplitude at −50 mV acquired in the absence (filled black squares) and presence (open red circles) of 3 μM KB−R7943 (mean ± SEM; *n* = 7 for each point). Current amplitude was measured at the end of voltage pulse at the level of −50 mV, and the absolute value of current amplitude is illustrated. (**C**) KB−R7943−mediated changes in *I*_Na(L)_ amplitude evoked by the envelope−of−tail test (mean ± SEM; *n* = 7 for each point). The relationships of the pulse duration versus the relative amplitude of *I*_Na(L)_ are illustrated with or without cell exposure to 3 μM KB−R7943. The relative amplitude appearing at the y−axis was measured in situations where *I*_Na(L)_ amplitude at the end of depolarizing pulse from −100 to −10 mV was divided by the tail current at the end of the voltage step taken following a return to −50 mV. The decaying rate of *I*_Na(L)_ evoked during the envelope−of−tail occurred in a single exponential function.

As the pulse duration applied for the elicitation of the inward current became prolonged, the NCX exchanging current seen in bullfrog atrial cells was noticed to be overly enhanced [1,3]. We thus continued to investigate if the presence of KB-R7943 could lead to any modifications in recovery from the decay of *I*_Na(L)_. The recovery from the current block was conducted with a two-step voltage clamp protocol in situations where the interval of depolarizing command pulses (i.e., conditioning pulse) was progressively prolonged. After each conditioning pulse, a clamp step repolarized back to the level of −50 mV for 20 ms was applied to evoke deactivating *I*_Na_ (Figure 5A). As demonstrated in Figure 5B, the relationship between the duration of conditioning pulse and amplitude of deactivating *I*_Na_ at the end of −50 mV with or without cell exposure to 3 μM KB-R7943 was afterwards established. Of note, the decaying time course of deactivating *I*_Na(L)_ became slowed with the presence of 3 μM KB-R7943, as demonstrated by a lengthening in decaying time constant of the current from 37 ± 3 to 48 ± 4 ms (*n* = 7, *p* < 0.05). Similarly, the relationship of the relative amplitude (i.e., *I*_Na(L)_ amplitude at −50 mV was divided by that at −10 mV) versus pulse duration (i.e., the envelope-of-tail test for *I*_Na(L)_) noticeably became decayed in an exponential fashion and is constructed in Figure 5C. Similarly, upon exposure to 3 μM KB-R7943, *I*_Na(L)_ evoked by the envelope-of-tail test was gradually decreased, as evidenced by an increase in decaying time constant of relative amplitude from 34 ± 3 to 48 ± 4 ms (*n* = 7, *p* < 0.05). The experimental results led us to reflect that the KB-R7943-mediated decrease in *I*_Na(L)_ appears to be independent of its suppressive actions on the activity of the NCX exchanging process.

### 2.6. Modification of Nonlinear Resurgent Na^+^ Current (I_Na(R)_) in Response to the Descending V_ramp_

**Figure 6:**
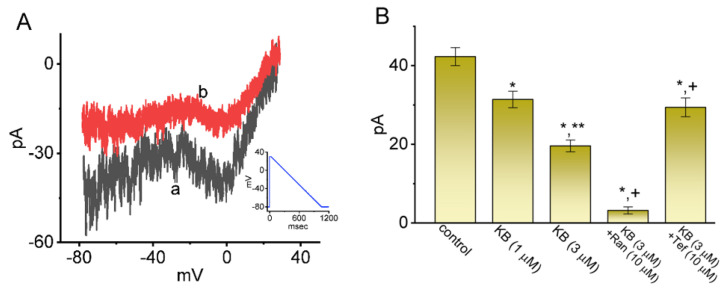
Inhibitory effect of KB−7943 on instantaneous resurgent Na^+^ current (*I*_Na(R)_) identified from GH_3_ cells. The experiments used to evoke non-linear *I*_Na(R)_ were designed to consist of a conditioning pulse step from −80 to +30 mV with a duration of 30 ms followed by a 1 s descending V_ramp_ from +30 to −80 mV (i.e., ramp pulse of −0.11 mV/ms). (**A**) Exemplar relationship of the current amplitude versus the membrane potential taken in the control period (a, black color) and during exposure to 3 μM KB−7943 (b, red color). Inset shows the voltage clamp protocol applied. (**B**) Summary graph disclosing effects of KB−7943 (KB, 1 or 3 μM), KB−7943 plus ranolazine (Ran), and KB−7943 plus tefluthrin (Tef) on the amplitude of *I*_Na(R)_ in response to the descending (repolarizing) V_ramp_ (mean ± SEM; *n* = 7 for each yellow bar). Each current amplitude evoked by the downsloping V_ramp_ was taken at the level of −5 mV. * Significantly different from control (*p* < 0.05), ** significantly different from KB-7943 (1 μM) alone group (*p* < 0.05), and ^+^ significantly different from KB−7943 (3 μM) alone group (*p* < 0.05).

The *I*_Na(R)_ has been identified in GH_3_ cells [27,34], and the magnitude of this current was also previously demonstrated to be intimately associated with high-frequency firing inherent in cerebellar Purkinje neurons [51,52,53,54]. This type of Na^+^ current is particularly unique, because it is not detectable until the membrane potential becomes repolarized below 0 mV. Alternatively, in addition to being activated by depolarizing voltage pulses rather than by repolarizing voltage steps, *I*_Na(R)_ was found to activate and decay more slowly than *I*_Na(T)_ [55]. As a result, *I*_Na(R)_ has been thought to help produce rapid depolarization immediately following an action potential; hence, it is suited either for cells that fire spontaneously at a higher firing rate, or to offer noise modulation in neurons with varying bursting firing [52,54,56]. In this regard, efforts were additionally made to see if the presence of KB-R7943 could exert any perturbations on such an instantaneous current evoked by the descending V_ramp_. As the whole-cell configuration was firmly made, we imposed the 30 ms depolarizing step from −80 to +30 mV followed by a descending (or repolarizing) V_ramp_ to −80 mV on the tested cell for a duration of 1 s. As demonstrated in Figure 6, the *I*_Na(R)_ magnitude in response to such voltage clamp protocol became overly reduced during cell exposure to KB-R7943. For example, within one minute of exposing cells to KB-R7943 at a concentration of 1 or 3 μM KB-R7943, *I*_Na(R)_ amplitude measured at −5 mV decreased from 42.3 ± 2.3 pA (*n* = 7) to 31.4 ± 2.1 pA (*n* = 7, *p* < 0.05) or 19.6 ± 1.5 pA (*n* = 7, *p* < 0.05), respectively. However, no clear modification in the voltage level (i.e., around −5 mV) for peak *I*_Na(R)_ elicitation was demonstrated with the presence of KB-R7943. Moreover, with continued exposure to 3 μM KB-R7943, the subsequent addition of ranolazine (10 μM, Ran) or Tef (10 μM) diminished or increased current amplitude to 3.2 ± 0.9 pA (*n* = 7, *p* < 0.05) or 29.4 ± 2.4 pA (*n* = 7, *p* < 0.05), respectively. Ran was earlier reported to be an inhibitor of *I*_Na(L)_ [25,37]. However, neither subsequent application of nimodipine (1 μM) nor CdCl_2_ (0.5 mM) had any effects on the KB-R7943-mediated decrease in *I*_Na(R)_. It can be interpreted to mean, therefore, that the exposure to KB-R7943 is capable of suppressing *I*_Na(R)_ magnitude during the descending V_ramp_ observed in these cells.

### 2.7. KB-R7943-Mediated Effect on Persistent Na^+^ Current (I_Na(P)_) Evoked by Sinusoidal Voltage Waveform

**Figure 7:**
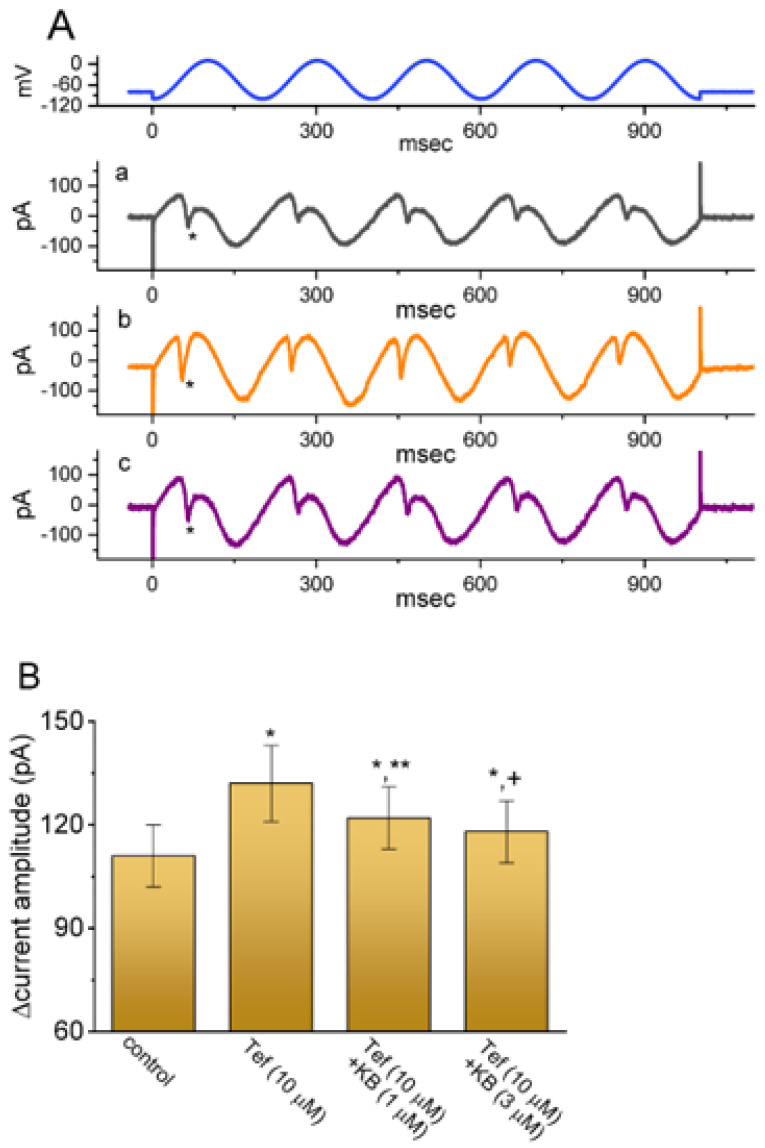
Attenuating effect of KB−R7943 on tefluthrin-stimulated *I*_Na(P)_ activated in response to sinusoidal voltage waveform. The tested cell was held at −80 mV, and the voltage clamp protocol designed to consist of sinusoidal voltages between −100 and 0 mV with a rate of 5 Hz for a duration of 1 sec was afterwards applied to it. (**A**) Exemplar current traces obtained in the control period (a, black color, i.e., neither Tef nor KB (KB−7943) was present), and during cell exposure to either 10 μM Tef (b, orange color) or 10 μM Tef plus 3 μM KB−R7943 (c, purple color). The asterisk in each panel indicates a transient inward deflection corresponding with the occurrence of *I*_Na(P)_ activated in response to sinusoidal voltage waveform. (**B**) Effect of tefluthrin (Tef) and Tef plus KB−R7943 (KB, 1 or 3 μM) on ∆current amplitude of *I*_Na(P)_ activated in response to sinusoidal voltage command (mean ± SEM; *n* = 7 for each brown bar). The absolute value of ∆current amplitude of *I*_Na(P)_ shown on the y-axis was measured when the difference between current amplitude at −60 mV and that at −30 mV was taken. * Significantly different from control (*p* < 0.05), ** significantly different from Tef (10 μM) alone group (*p* < 0.05), ^+^ significantly different from Tef (10 μM) plus KB (1 μM) group (*p* < 0.05).

There is growing evidence to show that a significant fraction of subthreshold or background Na^+^ currents is functionally active in varying types of excitable cells [20,22,24,27,57,58,59,60,61]. Recent investigations have also demonstrated possible modifications of sinusoidal voltage wave on membrane ionic currents [62,63,64,65]. For these reasons, efforts were further given to answer the question of whether *I*_Na(P)_ can be susceptible to adjustments by sinusoidal voltage waveform or whether or how sinusoidal voltage-induced *I*_Na(P)_ can be perturbed by adding KB-R7943. An example of a KB-R7943-mediated effect on sinusoidal waveform-activated *I*_Na(P)_ (transient inward deflection indicated in asterisk) seen in GH_3_ cells is illustrated in Figure 7A. It needs to be mentioned that with cell exposure to Tef (10 μM), the difference (i.e., ∆amplitude) taken between current amplitude taken at −60 mV and that at −30 mV was effectively enhanced. Moreover, the subsequent application of KB-R7943 (1 or 3 μM), still in the presence of Tef, could attenuate its stimulation of *I*_Na(P)_ activated by sinusoidal voltage waveform (Figure 7B).

### 2.8. Docking Prediction of hNa_V_1.7 and KB-R7943

**Figure 8:**
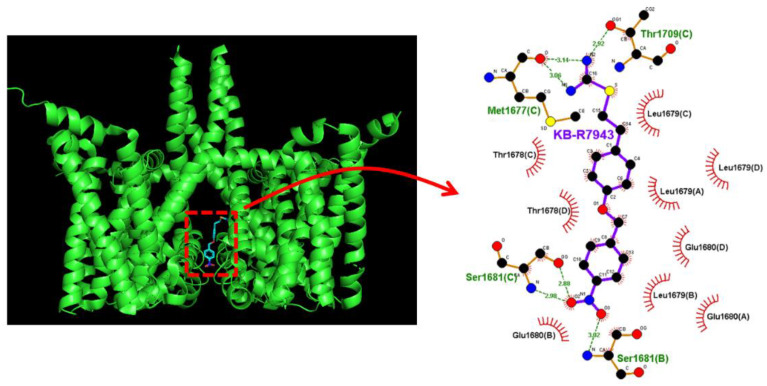
Molecular docking of the hNa_V_1.7 channel and the KB-R7943 molecule. The chemical structure of KB-R7943 was acquired from PubChem (compound CID: 9823846), whereas the protein structure of hNa_V_1.7 was from RCB PDB (ID: 5EK0). We docked the KB-R7943 molecule to hNa_V_1.7 with the help of PyRx software (http://pyrx.sourceforge.io/) (accessed on 16 September 2022), and the diagram of the interaction between the hNa_V_1.7 channel and the KB-R7943 molecule was then generated from LigPlot^+^ (http://www.ebi.ac.uk/thornton-srv/software/LIGPLOT/) (accessed on 16 September 2022). Note that the red arcs on which red small bars are faced and radiated toward the ligand (i.e., KB-R7943 molecule) represent the hydrophobic interactions, while green dotted lines residing in amino acid residue (i.e., Ser1681(B), Ser1681(C), Thr1709(C) and Met1877(C)) show the formation of hydrogen bonds.

In this study, we further explored how the protein of the hNa_V_1.7 channel could be appropriately docked with KB-R7943 with the help of PyRx software. The protein structure of hNa_V_1.7 was derived from RCB PDB (ID: 5EK0). The predicted docking sites of the KB-R7943 molecule with which the amino acid residues can interact are presented in Figure 8**.** Accordingly, it is important to mention that the KB-R7943 molecule may form hydrophobic contacts with certain amino acid residues, including Thr1678(C), Thr1678(D), Leu1679(A), Leu1679(B), Leu1679(C), Leu1679(D), Glu1680(A), Glu1680(B), and Glu1680(D). The atom in the KB-R7943 molecule also has the formation of hydrogen bonds with residue Met1677(C) or Thr1709(C) with an estimated distance of 3.06 and 3.14 Å or 2.92 Å, respectively, and has the formation of hydrogen bonds with residue Ser1681(B) or Ser1681(C) with a distance of 3.02 Å or 2.98 and 2.88 Å, respectively. Therefore, based on the Na_V_1.7 protein sequence (GenBank: ASY-04966.1), the inactivation gate of the channel was found to be located at the residue positions ranging between 1459 and 1462, which are noticeably adjacent to the docking sites of the KB-R7943 channel. The results regarding molecular docking thus enable us to propose that the KB-R7943 molecule can appropriately dock to the transmembrane segment (position: 1665–683) of the hNa_V_1.7 channel (PDB: 5EK0). Moreover, the binding affinity for molecular docking was estimated to be −7.7 kcal/mol. As a result, in combination with the electrophysiological results described above, such molecular docking might support the notion that KB-R7943 can have a substantial impact on the magnitude and/or gating kinetics of I_Na_.

### 2.9. Docking Prediction of hNa_V_1.2 and KB-R7943

**Figure 9:**
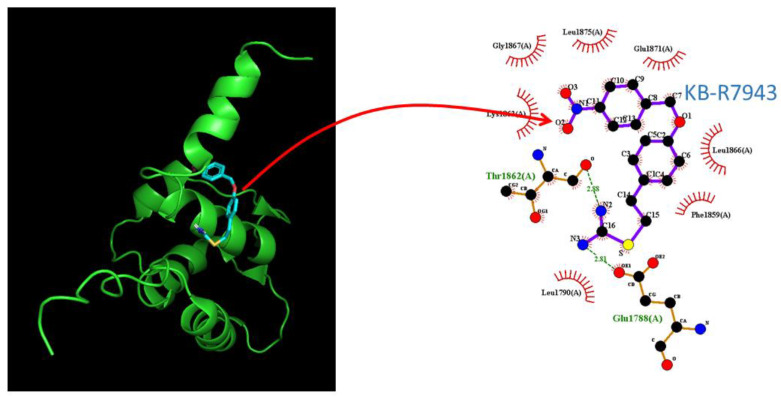
Molecular docking of the hNa_V_1.2 channel and the KB-R7943 molecule. The chemical structure of KB-R7943 was taken from PubChem (compound CID: 9823846), whereas the protein structure of hNa_V_1.2 was from RCB PDB (ID: 2KAV). We docked the KB-R7943 molecule to hNa_V_1.2 with PyRx, and diagram of the interaction between the hNa_V_1.2 channel and the KB-R7943 molecule was then generated from LigPlot^+^. Similar to those on the right side of Figure 8, the red arcs on which red small bars are faced and radiated toward the KB-R7943 molecule represent the hydrophobic interactions, whereas green dotted lines in amino acid residue (i.e., Glu1788(A) and Thr1862(A)) indicate the formation of hydrogen bonds.

It has been previously demonstrated that pituitary GH_3_ cells could express the mRNA transcripts for the α-subunit of Na_V_1.1, Na_V_1.2, Na_V_1.3, and Na_V_1.6, as well as β1- and β3subunits of Na_V_ channels [19]. Therefore, we further investigated how the protein of hNa_V_1.2 could be docked by KB-R7943 with PyRx software. The docking sites of the KB-R7943 molecule were shown in Figure 9. Notably, as it is docked to hNa_V_1.2, KB-R7943 can form hydrogen bond with residues Glu1788(A) and Thr1862(A) with distances of 2.81 and 2.88 Å, respectively. Furthermore, KB-R7943 can form hydrophobic contacts with several residues, including Glu1788(A), Leu1790(A), Phe1859(A), Lys1863(A), Leu1866(A), Gly1867(A), Glu1871(A), and Leu1875(A). This prediction thus reflects that KB-R7943 can bind to the amino acid residues of the hNa_V_1.2 channel with an estimated binding affinity of −6.3 kcal/mol. Such predicted interactions could potentially affect the KB-R7943-mediated change in *I*_Na_ described above.

## 3. Discussion

The important findings in this study are as follows. (a) In pituitary GH_3_ lactotrophs, the presence of KB-R7943, thought to suppress the activity of the NCX exchanging process, could suppress *I*_Na_ in concentration-, time-, and voltage-dependent manners. (b) The estimated IC_50_ values required for KB-R7943-inhibited the amplitude of *I*_Na(T)_ and *I*_Na(L)_ were distinguishable (i.e., 11 and 0.9 μM, respectively). (c) Either benzamil or amiloride, also known to be inhibitors of the NCX exchanging current, was found to suppress *I*_Na(L)_ amplitude. (d) The steady-state *I-V* relationship of *I*_Na(T)_ remained unaltered in the KB-R7943 presence. (e) The strength (i.e., ∆area) of instantaneous *I*_Na(W)_ activated by abrupt ascending V_ramp_ became depressed by adding KB-R7943; however, with continued exposure to this compound, further addition of deltamethrin (DLT) or tefluthrin (Tef) effectively attenuated the KB-R7943-mediated decrease in V_ramp_-induced *I*_Na(W)_. (f) As the duration of depolarizing pulse was prolonged, the amplitude of *I*_Na(L)_ became diminished as a function of time in an exponential fashion; furthermore, the exposure to KB-R7943 decreased *I*_Na(L)_ amplitude. (g) The presence of this compound suppressed resurgent Na^+^ (*I*_Na(R)_) evoked by the repolarizing V_ramp_; moreover, further exposure either to ranolazine (Ran) or Tef, respectively, diminished or attenuated the KB-R7943-mediated decrease in *I*_Na(R)_. (h) The persistent *I*_Na_ (*I*_N(P)_) evoked by sinusoidal voltage waveform was increased by adding Tef; moreover, the subsequent application of KB-R7943 could attenuate such Tef-stimulated *I*_Na(P)_. (i) The molecular docking of KB-R7943 to hNa_V_1.2 or hNa_V_1.7 was predicted because of the presumed formation of both hydrophobic contacts and hydrogen bonds. Taken together, the experimental results therefore allow us to reflect that, in concert with the inhibitory effect on the reverse mode of the NCX exchanging process in varying cell types [6,7,40,46,61,66,67,68,69], KB-R7943-mediated perturbations in *I*_Na(T)_, *I*_Na(L)_, *I*_Na(W)_, *I*_Na(R)_, and *I*_Na(P)_ tend to be independent of KB-R7943’s suppressive action on the activity of the NCX exchanging process. Therefore, these actions are anticipated to participate in potential modifications of the functional activities (e.g., various firing patterns) in electrically excitable cells.

In this study, cell exposure to KB-R7943 was capable of suppressing the amplitude of *I*_Na(T)_ as well as shortening the inactivation time course of the current activated by abrupt depolarizing voltage command. A concentration-dependent inhibition of *I*_Na(T)_ or *I*_Na(L)_, with effective IC_50_ values of 11 or 0.9 μM, respectively, was also obtained. The *K*_D_ value evaluated from quantitative estimate of the inactivation time course of *I*_Na(T)_ was also yielded to 0.97 μM (in the Supplementary Information), a value that was noted to be similar to the IC_50_ value required for its suppression of *I*_Na(L)_, but not for *I*_Na(T)_. Pertinent to this reaction scheme is thus that the open-blocked Na_V_ channels tend to be not closed unless the KB-R7943 molecule dissociates from the binding site(s). Meanwhile, the time-dependent block caused by this compound suggests that it preferentially binds to and blocks the open/inactivated state (conformation) of the Na_V_ channels, thereby leading to a destabilization in open conformation [16]. Moreover, although the steady-state *I-V* relationship of *I*_Na(T)_ was unaffected during the presence of KB-R7943, this compound diminished the strength of *I*_Na(W)_ or *I*_Na(R)_ evoked by respective ascending or descending V_ramp_. Therefore, whatever ionic mechanisms are involved, the effectiveness of this compound in the perturbations of *I*_Na_ described herein appear to be independent of an interaction with the NCX exchanging current; hence, it could be viewed to be an additional yet important factor for influencing functional activities of endocrine, neuroendocrine, or neuronal cells (e.g., membrane excitability).

Previous reports have demonstrated that with the increasing duration of the depolarizing pulse, either the magnitude of the NCX exchanging current in frog atrial cells or the Ca^2+^-activated nonselective cationic current in pituitary cells was progressively increased [1,44]. In contrast, the deactivating *I*_Na(L)_ presented herein was noted to decrease in an exponential fashion as the duration of depolarizing voltage step was prolonged when cells were exposed to Ca^2+^-free Tyrode solution (Figure 5). The *I*_Na(L)_ activated by the envelope-of-tail methods showed a decay in a time-dependent manner. Moreover, as the measuring electrode was filled with an internal solution containing a high concentration of EGTA (10 mM), the ability of KB-R7943 to suppress *I*_Na(T)_ and *I*_Na(L)_ still remained effective. It is therefore reasonable to assume that the reduction in *I*_Na(L)_ produced by KB-R7943 is unlikely to be associated with the suppression of NCX exchanging activity, although the NCX exchanging process was earlier demonstrated to be functionally expressed in endocrine or pituitary cells [4,39,41,42,43]. KB-R7943 has been reported to suppress the reverse mode of NCX1 with effective IC_50_ of 1.2–2.4 μM [7]. Moreover, in cultured hippocampal neurons, KB-R7943 exhibits neuroprotection from glutamate-induced excitotoxicity by blocking NMDA receptor-mediated activity (IC_50_ = 13.4 μM) as well as by inhibiting complex I in the mitochondrial respiratory chain (IC_50_ = 11.4 μM) [8]. Under this scenario, the inhibitory effect of this compound on the magnitude and gating of *I*_Na_ could be of mechanistic, pharmacological, or even clinical relevance [15,16,70].

It also needs to be mentioned that either instantaneous *I*_Na(W)_ or *I*_Na(R)_ evoked by the rising or downsloping (or repolarizing) V_ramp_, respectively, can be susceptible to being suppressed by adding this compound. Although the overall steady-state *I-V* relationship of *I*_Na(T)_ remained unaffected in the presence of KB-R7943, the addition of this compound also had the propensity to suppress the *I*_Na(W)_’s strength as well as the *I*_Na(R)_ magnitude activated by V_ramp_. Continued exposure to KB-R7943, but still in the presence of Tef or DLT, could reverse the stimulation of *I*_Na(W)_; moreover, either the subsequent addition of Ran or Tef diminished or attenuated, respectively, the KB-R7943-mediated decrease in *I*_Na(R)_. Previous reports have shown that the extent of *I*_Na(W)_’s strength is linked to the magnitude of background or residual steady Na^+^ currents in many excitable cells [47,48,50,71]. Meanwhile, *I*_Na(R)_’s magnitude has also been demonstrated to be associated with high-frequency or varying burst firing of action potentials in excitable cells (e.g., cerebellar Purkinje neurons) [51,52,53,54,55,56,72]. Moreover, the *I*_Na(P)_ magnitude induced by sinusoidal voltage waveform was also augmented by adding Tef; of note, the further addition of KB-R7943 could attenuate Tef-augmented *I*_Na(P)_. The docking results shown herein predicted an interaction of the hNa_V_1.2 or hNa_v_1.7 channel and the KB-R7943 molecule. This study also showed the ability of benzamil or amiloride to suppress *I*_Na(L)_ magnitude. As such, the modifications by KB-R7943 of *I*_Na(W)_, *I*_Na(R)_, and *I*_Na(P)_ demonstrated herein are important and should not be underestimated. Caution thus needs to be exercised in attributing the action of KB-R7943 or other relevant compounds on the functional activities of excitable cells solely to their suppression in the activity of the NCX exchanging process [6,7,40,46,61,66,67,69,73].

## 4. Materials and Methods

### 4.1. Chemicals, Drugs, and Reagents used in this Work

KB-R7943 (2-[2-[4-(4-nitrobenzyloxy)phenyl]ethyl]isothiourea, 2-[4-[(4-nitrophenyl)methoxy]phenyl]ethyl ester carbamimidothioic acid, C_16_H_17_N_3_O_3_S·CH_3_SO_3_H) was supplied by Cayman (Excel Biomedical, Tainan, Taiwan). Nimodipine, tefluthrin (Tef), tetraethylammonium chloride (TEA), and tetrodotoxin (TTX) were acquired from Sigma (Merck, Taipei, Taiwan); benzamil and amiloride from Tocris (Union Biomed Inc., Taipei, Taiwan); and deltamethrin (DLT, deltamethrin) from MedChemExpress (Asia Bioscience, Taipei, Taiwan). For long-term storage, the stock solution of KB-R7943 was stored at −20 °C in the dark. Culture media (e.g., Ham’s F-12 medium), fetal calf serum, horse serum, L-glutamine, and trypsin/EDTA were supplied by HyClone^TM^ (Genchain, Kaohsiung, Taiwan). Other reagents or chemicals were of the highest purity available from commercial sources. Freshly prepared ultrapure water provided by APS Water Services Inc. (Van Nuys, CA, USA) was used in all experiments.

The ionic compositions of the external solution (i.e., HEPES-buffered normal Tyrode solution) were as follows (in mM): NaCl 136.5, CaCl_2_ 1.8, KCl 5.4, MgCl_2_ 0.53, glucose 5.5, and HEPES 5.5 (pH 7.4 adjusted by adding NaOH). To measure ionic currents flowing through K^+^ currents, the patch electrode was filled with the internal solution comprising (in mM) K-aspartate 130, KCl 20, KH_2_PO_4_ 1, MgCl_2_ 1, EGTA 0.1, Na_2_ATP 3, Na_2_GTP 0.1, and HEPES 5 (pH adjusted with KOH). To record variable types of voltage-gated Na^+^ current (*I*_Na_), K^+^ ions inside the pipette solution were replaced with equimolar Cs^+^ ions, and the pH was titrated to 7.2 with CsOH. In some experiments designed to highly buffer intracellular Ca^2+^, the internal pipette solution contained EGTA at a concentration of 10 mM. All solutions were prepared by using deionized water which was produced by a Milli-Q^®^ water purification system (Shih Jhih Technology, Tainan, Taiwan).

### 4.2. Cell Preparations

GH_3_, a clonal cell line originally derived from a rat prolactin-secreting pituitary tumor, was acquired from the Bioresources Collection and Research Center (BCRC-60015; Hsinchu, Taiwan). Briefly, cells were grown in Ham’s F-12 medium supplemented with 15% heat-inactivated horse serum (*v*/*v*), 2.5% fetal calf serum (*v*/*v*), and 2 mM L-glutamine in a humidified environment of 5% CO_2_/95% air [10,44]. Under the experimental conditions presently used, cells remained 80–90% viable for at least two weeks. Subcultures were made by trypsinization (0.025% trypsin solution (HyClone^TM^) containing 0.01% sodium *N*,*N*-diethyldithiocarbamate and EDTA).

### 4.3. Electrophysiological Measurements

Shortly before the experiments, cells were carefully detached from culture dishes, and an aliquot of cell suspension was transferred to a homemade chamber and allowed to settle to the bottom. The chamber was firmly positioned on the stage of a CKX-41 inverted microscope (Olympus; Yuan-Li, Kaohsiung, Taiwan), and cells were immersed at room temperature (20–25 °C) in normal Tyrode solution, the ionic compositions of which are described above. Patch clamp recordings in the whole-cell configuration were performed with the help of either an RK-400 (Bio-Logic, Claix, France) or an Axopatch-200B patch amplifier (Molecular Devices; Bestogen Biotech, New Taipei City, Taiwan) [10,22,74]. Patch electrodes with tip resistances of 3–5 MΩ were made of Kimax^®^-51 glass capillaries (#34500-99; Kimble^®^; Dogger, New Taipei City, Taiwan) by using a PP-830 vertical puller (Narishige; Major Instruments, New Taipei City, Taiwan), and then fire-polished with an MF-83 microforge (Narishige). The junction potential, which occurred due to different compositions between extracellular and intracellular solutions, was zeroed shortly before GΩ-seal formation, and the whole-cell data were then corrected.

### 4.4. Whole-Cell Data Recordings with Patch-Clamp Technique

The signal output (i.e., potential and current traces) was monitored on an HM-507 oscilloscope (Hameg, East Meadown, NY, USA) and digitally stored online in an Acer SPIN-5 laptop computer (Yuan-Dai, Tainan, Taiwan) at 10 kHz or more through the Digidata^®^ 1440-A interface (Molecular Devices, Sunnyvale, CA, USA). During the measurements, the latter device was controlled by pCLAMP^TM^ 10.6 software (Molecular Devices) run under Microsoft^®^ Windows^TM^ 7 (Redmond, WA, USA). We low-pass filtered current signals at 2 kHz with an FL-4 four-pole Bessel filter (Dagan, Tainan, Taiwan) before they were digitized. Through digital-to-analog conversion, manifold pCLAMP-generated voltage protocols (i.e., rectangular, ramp, or sinusoidal waveforms) were tailored to determine the nonlinear properties of either window Na^+^ current (*I*_Na(W)_), resurgent Na^+^ current (*I*_Na(R)_), or persistent Na^+^ current (*I*_Na(P)_). After the signals were digitally stored, we analyzed them offline by using manifold analytical tools that include LabChart^TM^ 7.0 program (AD Instrument, KYS Technology, Tainan, Taiwan), OriginPro^®^ 2022b (Microcal; Scientific Formosa, Kaohsiung, Taiwan), and custom macro procedures built under Microsoft^®^ Excel^®^ 2021 (Redmond, WA, USA).

### 4.5. Data Analyses

To assess sigmoidal dose-dependent inhibition of KB-R7943 on the peak or transient *I*_Na_ (*I*_Na(T)_) and sustained or late *I*_Na_ (*I*_Na(L)_) of the voltage-gated Na^+^ current (*I*_Na_), we placed GH_3_ cells in Ca^2+^-free Tyrode solution, and the measuring electrode used presently was filled up with an internal solution containing Cs^+^. To measure *I*_Na(T)_ and *I*_Na(L)_, we voltage-clamped each examined cell at −100 mV, and the depolarizing voltage command steps to −10 mV for a duration of 30 ms were delivered at a rate of 0.1 Hz. The *I*_Na(T)_ or *I*_Na(L)_ amplitudes taken at the start or end-pulse of each depolarizing pulse were respectively measured during the control period (i.e., KB-R7943 was not present) and with cell exposure to different concentrations of KB-R7943 (0.3–30 μM). The IC_50_ value required for KB-R7943-mediated inhibition of *I*_Na(T)_ or *I*_Na(L)_ observed in GH_3_ cells was thereafter optimally determined by using a modified Hill function, as follows:

Relative amplitude={[KB-R7943]−nH×(1−a)}{[KB-R7943]−nH+IC50−nH}+a

In this equation, IC_50_ is the concentration required for 50% inhibition, n_H_ is the Hill coefficient, and [KB-R7943] is the KB-R7943 concentration applied. Maximal inhibition (i.e., 1−a) was approximated from this equation. This formula could converge in an appropriate way to give the best-fitting sigmoidal line and the reliable parameter estimates (e.g., IC_50_).

### 4.6. Curve-Fitting Approximations and Statistical Analyses

Linear or nonlinear curve fitting (e.g., sigmoidal or exponential curve) to each data set was approximated with the least-squares minimization procedure by using manifold maneuvers, which include the “Solver” function embedded in Excel^®^ 2016 (Microsoft^®^), OriginPro^®^ 2022b program (OriginLab^®^; Scientific Formosa, Kaohsiung, Taiwan) and MATLAB^®^ 7.0 (I-Planet Information, Tainan, Taiwan). The averaged results are presented as the mean ± standard error of the mean (SEM) with independent sampling sizes (*n*) indicating cell numbers from which the observations were properly collected. For two different groups, we used the paired or unpaired Student’s *t*-test. To evaluate the differences among more than two groups (e.g., intertreatment differences), we utilized one-way analysis of variance (ANOVA-1 or ANOVA-2) followed by the post hoc Fisher’s least-significance difference method. Statistical analyses were made using the SPSS 20 software package (AsiaAnalytics, Taipei, Taiwan). Statistical significance was determined at a *p*-value of < 0.05 (^*^, ^**^, or ^+^ are indicated in the figures).

## Data Availability

The original data are available upon reasonable request to the corresponding author.

## Supplementary Materials

The following supporting information can be downloaded at: https://www.mdpi.com/article/10.3390/ijms24021805/s1.

## Abbreviations

DLT	deltamethrin
*I* *-V*	current versus voltage
IC_50_	the concentration required for 50% inhibition
*I*_Na_	voltage-gated Na^+^ current
KB	KB-R7943 (2-[2-[4-(4-nitrobenzyloxy)phenyl]ethyl]isothiourea)
*I*_Na(L)_	late Na^+^ current
*I*_Na(R)_	resurgent Na^+^ current
*I*_Na(T)_	transient Na^+^ current
*I*_Na(W)_	window Na^+^ current
*K*_D_	dissociation constant
NCX exchanger	Na^+^-Ca^2+^ exchanger
Ran	ranolazine
SEM	standard error of mean
τ_inact(S)_	time constant in the slow component of current inactivation
TEA	tetraethylammonium chloride
Tef	tefluthrin
TTX	tetrodotoxin
V_ramp_	ramp voltage
