# Supplementary material for: Inhibition of Voltage-Gated Na+ Currents Exerted by KB-R7943 (2-[2-[4-(4-nitrobenzyloxy)phenyl]ethyl]isothiourea), an Inhibitor of Na+-Ca2+ Exchanging Process
Source: Int J Mol Sci. 2023 Jan 16;24(2):1805. doi: 10.3390/ijms24021805 (PMC9864174; doi:10.3390/ijms24021805)
Supplement: Supplementary file 1 [file ijms-24-01805-s001.zip › ijms-2086908-supplementary.pdf]

Supplementary Information

Kinetic studies of KB-R7943-mediated inhibition of I_{Na} measured from pituitary GH_3 cells

To provide quantitative estimate for KB-R7643-induced block of I_{Na} activated in response to short depolarizing pulse, we further evaluated the time-dependent inactivation indicating the block of I_{Na} observed in these cells. We fitted the time courses of current inactivation during cell exposure to different concentrations of KB-R7943 by a two-exponential function, i.e., fast and slow inactivation time course of the current. The concentration dependence of I_{Na} inactivation elicited by abrupt depolarizing pulse from -100 to -10 mV was examined. The results showed that with the presence of KB-R7943, it produced a concentration-dependent raise in the rate (1/◎$_{inact(S)}$) of I_{Na} in response to rapid membrane depolarization.

The inhibitory action of KB-R7943 on the slow time course of I_{Na} inactivation measured from GH_3 cells is reasonably explained by state-dependent block that preferentially binds to the open/inactivated state of the Na_V channel. A minimal kinetic scheme was hence derived as the following:

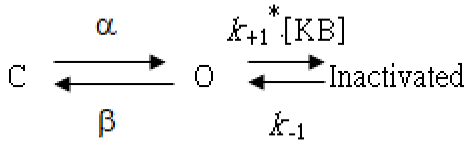

where [KB] is the KB-R7943 concentration applied; ◎ or ◎ is the voltage rate constant for the opening or closing of Na_V channels, respectively; k_{+1}^{*} or k_{-1} is the rate constant for blocking and unblocking produced by the presence of KB-R7943; and, C, O, or

Inactivated shown in the scheme points out the close, open, or inactivated state, respectively.

The blocking (i.e., on) and unblocking (i.e., off) rate constants, k_{+1}^{*} and k_{-1}, were determined from the slow component in inactivation time constants (i.e., ◎$_{inact(S)}$) of I_{Na} obtained in different concentrations of KB-R7943. These rate constants (1/◎$_{inact(S)}$) were then allowed to be computed using the relation (**Supplementary Figure 1**):

$$1/\tau_{inact(S)} = k_{+1}^{*} \times [KB] + k_{-1}$$

where k_{+1}^{*} and k_{-1} are respectively approximated from the slope and from the *y*-axis intercept at [KB] = 0 of the linear regression where the reciprocal time constants (1/◎$_{inact(S)}$) versus the KB-R7943 concentrations were interpolated, and [KB] is the KB-R7943 concentration given.

According to the first-order binding scheme, the relationship between 1/◎$_{inact(S)}$ and [KB] became linear (**Supplementary Figure 1**). The blocking and unblocking rate constants were estimated to be 0.252 msec^{-1}◎M^{-1} and 0.245 msec^{-1}, respectively; hence, the results yielded the value of dissociation constant (K_D = k_{-1}/k_{+1}) of 0.97 ◎M. The value is noticeably close to effective IC_{50} required for KB-R7943-mediated decrease of $I_{Na(L)}$ in GH_3 cells.

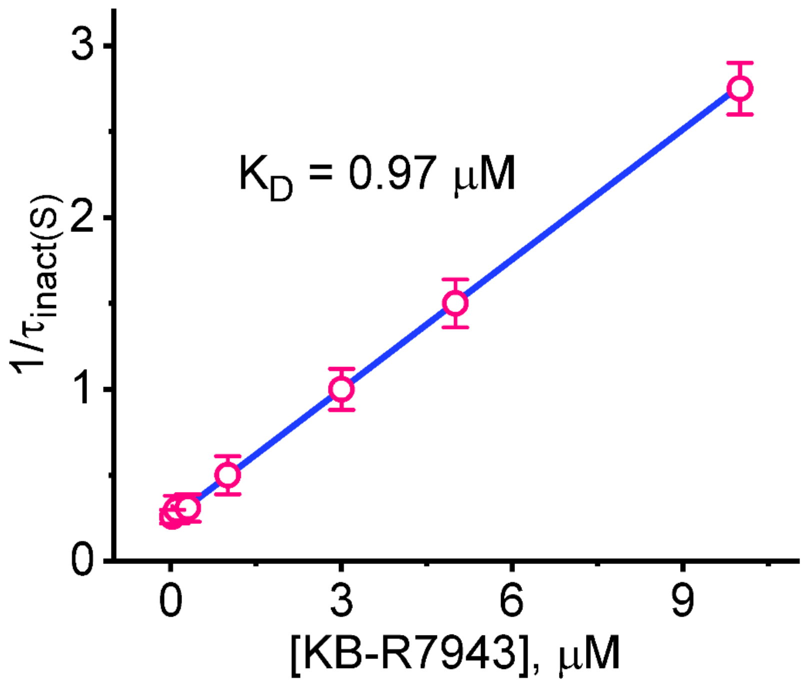

Supplementary Figure S1. Concentration-dependent raise of the rate constant ($◎_{inact(S)}$) of I_{Na} inactivation observed in GH_3 cells. The reciprocal of time constant (i.e., $1/◎_{inact(S)}$) of current inactivation versus the KB-R7943 concentration is illustrated. Data points appearing in open red circles were fitted by a linear regression (blue line), reflecting that there is molecularity of one. Each point represents the mean ± SEM (n = 8).
